# First Report of Histotripsy-Induced Survival Benefit in Murine Glioblastomas

**DOI:** 10.3390/cancers18040622

**Published:** 2026-02-13

**Authors:** Sarah Duclos, Tarana Parvez Kaovasia, Adam Fox, Ashley Cornett, Aditya S. Pandey, Zhen Xu

**Affiliations:** 1 Department of Biomedical Engineering, University of Michigan, Ann Arbor, MI 48109, USA; tarank@umich.edu (T.P.K.); arieck@umich.edu (A.C.); zhenx@umich.edu (Z.X.); 2 Department of Neurosurgery, University of Michigan, Ann Arbor, MI 48109, USA; foxadam@umich.edu (A.F.); adityap@med.umich.edu (A.S.P.); 3 Department of Radiology, University of Michigan, Ann Arbor, MI 48109, USA

**Keywords:** glioblastoma, murine model, transcranial histotripsy

## Abstract

**Background:** Glioblastoma (GBM) is a lethal, highly invasive, and recurrent brain tumor. Standard treatment combines maximal surgical resection, radiation, and chemotherapy; however, such approaches are often infeasible for tumors in eloquent brain regions. **Objective:** Histotripsy is a noninvasive, nonthermal ultrasound-based mechanical ablation modality that employs focused acoustic energy for targeted tissue destruction. This study aimed to investigate the feasibility, safety, and therapeutic effect of a one-time transcranial histotripsy treatment in a pre-clinical murine GBM model. **Methods:** GL261 GBM cells were orthotopically implanted into C56BL/6 mouse brains. Transcranial histotripsy was performed using a stereotactically guided 2 MHz transducer targeting either lower (25%) or higher (75%) tumor volume, with 5 or 10 pulses per location (PPL) administered. Tumor growth and cerebral injury were monitored with weekly magnetic resonance imaging (MRI) following treatment. At the study endpoint, hematoxylin and eosin (H&E) histology assessed residual tumor burden and histotripsy-induced tissue changes. **Results:** Mice receiving 5 PPL high-percent treatment (>30 sites) showed a statistically significant median survival extension of 5 days (18.5%) compared to untreated controls. MRI demonstrated marked tumor volume reduction in the high-percent treatment group at week 4, while H&E confirmed increased tumor necrosis and cellular damage in the treated cohort. **Conclusions:** Single-session, incisionless transcranial histotripsy was well tolerated and conferred mild yet meaningful survival advantages in this GBM model. These results support ongoing exploration of histotripsy, alone or in combination with existing therapies, for safe and effective treatment of challenging brain tumors.

## Simple Summary

Histotripsy is a noninvasive soft tissue ablation technique that uses high-pressure ultrasound to generate precise regions of cellular destruction within tumors while sparing surrounding healthy tissue. This study evaluated the survival benefit of a single transcranial histotripsy treatment in glioblastoma-bearing mice using a raster-scanning pattern with varying numbers of histotripsy pulses. Histotripsy resulted in an 18.5% increase in survival compared with untreated controls and was well tolerated with no significant acute or chronic adverse effects. These findings support further investigation of histotripsy as a potential therapeutic approach for brain tumors.

## 1. Introduction

Primary malignant brain tumors are one of the most devastating diseases in the United States, with approximately 17,000 deaths per year and an average 5-year survival rate of 35% [1]. Glioblastoma (GBM) is the most common type of primary brain cancer and has a particularly poor prognosis due to its aggressive growth near critical brain structures, often preventing maximal surgical resection [2]. Despite significant efforts to improve the outcomes of GBM patients over the last few decades, there has been no substantial improvement in the average survival rate [3]. Current standard-of-care treatment for malignant brain tumors includes maximal safe surgical resection, when possible, followed by concurrent chemotherapy and radiation [4]. However, the treatment of recurrent brain tumors is even more challenging, as there is no effective standard-of-care therapy for such lesions [5].

Maximal surgical resection is not possible in all regions of the brain and can be associated with resection-associated complications, including surgical site infection, bleeding, cerebral edema, and neurological deficits [4]. Furthermore, safe craniotomy-based surgical resection requires neurosurgical expertise as well as costly surgical equipment, including image guidance and magnetic resonance imaging/operating room (MRI/OR) technologies [6]. Despite these limitations, standard resection surgery remains the paradigm, as the extent of resected tumor is positively correlated to survival rate [7,8,9]. There is a need for improved minimally invasive methods to treat brain tumors, reducing the necessity for extensive surgical resection while maximizing the safe and effective elimination of brain tumor cells in real-time. Conventional surgical resection relies on direct physical removal of tumor tissue and is fundamentally limited by line-of-sight access, brain shift, and the need to preserve surrounding eloquent structures. Thermal and radiation-based adjuncts, while less invasive, depend on heat deposition or ionizing radiation and are constrained by off-target tissue injury and cumulative dose limits [10]. In contrast, histotripsy operates through a nonthermal, cavitation-mediated mechanism, enabling focal tissue disruption without reliance on thermal diffusion, ionizing radiation, or surgical access.

Considering this need, histotripsy has the potential to noninvasively treat brain tumors. This developing technology uses nonionizing and nonthermal focused ultrasound therapy. Histotripsy is FDA-approved for the treatment of liver tumors [11] and is gaining attention for its use as a transcranial application to treat brain tissue without the need for craniotomy. By using extremely high-pressure ultrasound pulses (>30 MPa) at a low duty cycle (<1%) to minimize tissue heating, histotripsy can precisely ablate soft tissue via mechanical cavitation with submillimeter precision [11,12,13]. Recently, histotripsy has been shown to be effective for ablating brain tissue through an intact skull in porcine tissue [14,15], human cadaver brain tissue [16], and murine brain tumor models [17,18,19]. In addition to brain tissue ablation, histotripsy has demonstrated transient blood–brain barrier (BBB) opening at the periphery of the ablation zone [18].

Previous studies have suggested that treating a sub-portion of the tumor with histotripsy may incite higher levels of anti-tumor immune effects, resulting in prolonged survival [20,21]. Given the consensus that maximal safe resection of brain tumors leads to survival benefit, we aimed to understand whether transcranial histotripsy could provide incisionless treatment of brain tumors [8,9]. This is the first study to examine animal survival as a function of the percentage of brain tumors targeted with a one-time stand-alone histotripsy treatment for two different pulses per location regimes (5 and 10 PPL).

## 2. Materials and Methods

### 2.1. Animals

The University of Michigan Institutional Animal Care Use Committee (IACUC) approved all animal experiments (IACUC # PRO00010789) on 18 April 2022. This study used wild-type C57BL/6 mice (000058 B6(Cg)-Tyr<c-2J>/J, Jackson Labs, 6–10 weeks old, *n* = 43 mice total). Mice were kept in a 12 h light/dark cycle with unrestricted access to food and water for the entirety of the experiments.

### 2.2. Tumor Implantations

GL261 cells were gifted from the Departments of Radiology and Surgery at the University of Michigan and were injected directly into the brains of mice using a stereotactic intracranial injection method [18]. Briefly, mice were initially anesthetized with 5% isoflurane and 1% oxygen and maintained at 2% isoflurane and 1% oxygen for the remainder of the procedure. After fur removal and skin sterilization, a 1 cm anterior–posterior incision was made, and a small area of periosteum was removed with a cotton tip to expose the skull. A 1 mm burr hole was drilled into the skull approximately 2 mm lateral and 1 mm anterior to the bregma in the right brain hemisphere. A stereotactic rodent brain injection system was used for tumor implantations, as described by Choi et al. [22]. The syringe and needle system (10 μL, Hamilton, Reno, NY, USA) was positioned 4 mm deep relative to the brain surface in the striatum and extracted 0.5 mm to create a pocket for the cell injection. Cells were suspended in 1 μL of plain Dulbecco’s Modified Eagle Medium (Gibco, Grand Island, NY, USA) at a concentration of 100,000 cells/μL. A total of 0.5 μL of the cell suspension was injected over one minute, and the needle was extracted slightly. The rest of the cell suspension (0.5 μL) was injected over 1 min, and the needle was extracted from the brain 7 min later in 0.5 mm/min increments until the needle cleared the surface of the brain. The injection site was plugged with bone wax, and the incision was secured with skin glue. Mice were administered carprofen (5 mg/kg subcutaneous) and placed in a recovery chamber for 20 min following the procedure. All mice regained normal behavior and movement within the 20 min recovery period.

### 2.3. Histotripsy

**Figure 1:**
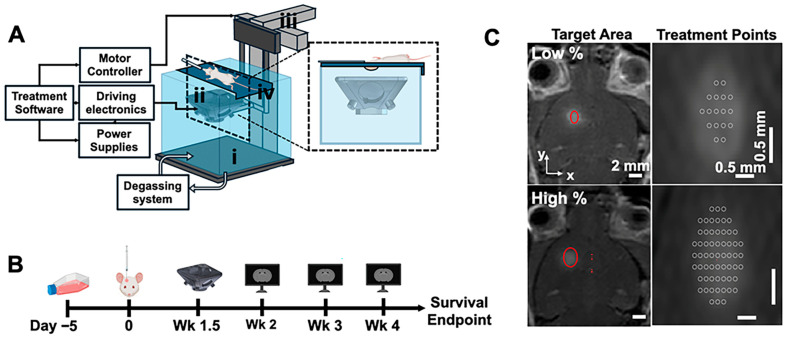
(**A**) Schematic of histotripsy system setup. The system comprised a degassed water bath (**i**), a 2 MHz, 5-element custom-built histotripsy array (**ii**), 3-axis motor positioning system to which the histotripsy array was attached (**iii**), circulating degassing system, motor controlling electronics, driving electronics for the histotripsy array, power supplies for the driving electronics, and custom treatment software for controlling the system components. The anaesthetized mouse was secured to a treatment platform (**iv**) such that only the shaved head was submerged in the water bath for ultrasound coupling. (**B**) Experimental timeline. GL261 cells were cultured for approximately 5 days prior to intracranial tumor cell implantations (Day 0). Histotripsy was performed 1.5 weeks (10–12 days) following tumor implantations. Tumor growth was monitored with MRI at weeks 2–4 (days 15–17, days 21–13, and days 26–28, respectively) following tumor implantations. Mice were monitored and weighed until they reached their survival endpoint, after which their brains were collected for histological analysis. (**C**) Low-percentage treatments (top row) targeted the tumor core, covering approximately 25% of the tumor (<30 total treatment locations). High-percentage treatments (bottom row) targeted approximately 75% of the tumor (>30 total treatment locations). Red circles indicate the targeted area in the tumor. Abbreviations: Wk, week.

Transcranial histotripsy was stereotactically performed with a custom-built 2 MHz, 5-element transducer (focal distance = 25 mm, aperture = 40.95 mm, f # = 0.6) (Figure 1A). Mice underwent pretreatment MRI to obtain T1-weighted MRI scans with gadolinium enhancement (ProHance, 5 mg/kg I.P.) for treatment planning. Mice were then placed on a treatment platform secured at the surface of a degassed water tank (37 °C, <15% dissolved oxygen), such that only the shaved head was submerged in water. The transducer was attached to a 3-axis motor positioner controlled by a custom-written MATLAB version 2024b (Natick, MA, USA) script and targeted the tumor via an MRI-based stereotactic targeting method with a raster scanning targeting pattern [22]. Briefly, using fiducial markers obtained from pretreatment MRI, the location of the transducer focus was co-localized with the center of the tumor with submillimeter accuracy [22].

A panel of parameters was tested with the 2 MHz transducer. Mice were treated at a pulse repetition frequency of 5 Hz with 5 or 10 histotripsy pulses per treatment location (PPL), targeting a lower or higher percentage of the tumor (Figure 1C). Lower-percentage treatments targeted the tumor core, covering approximately 25% of the tumor (<30 total treatment locations). Higher-percentage treatments targeted approximately 75% of the tumor (>30 total treatment locations). Five and ten PPL were selected based on prior studies demonstrating effective ablation of murine brain tissue with minimal hemorrhagic effects [17]. The 10 PPL protocol was used for larger tumors to account for increased mechanical heterogeneity, including necrotic cores, stiffer peripheral regions, and increased tumor cell density and interstitial fluid pressure [23,24]. This approach aimed to achieve consistent tumor damage across all treatment locations. Mice were randomly assigned to groups upon confirmation of tumor growth via MRI within 2 days of treatment.

Treatment locations were confined to a single axial plane passing through the center of the tumor and delivered in a raster scan pattern with an interpoint spacing of 0.20 mm in both the x and y directions (free-field bubble cloud size = 0.7 × 1.3 × 0.6 mm in *z*, *x*, and *y* directions, respectively). A derated focal pressure of approximately 36 MPa was used for all experiments, determined via sub-aperture, sub-cavitation threshold free-field water bath measurements using a fiber optic hydrophone (HFO 690, Onda, Sunnyvale, CA, USA). The 5 PPL group (*n* = 9 control, *n* = 9 low-percent, *n* = 9 high-percent) and 10 PPL group (*n* = 6 control, *n* = 6 low percent, *n* = 6 high percent) were analyzed as independent studies because treatments were applied at different stages of tumor growth; all comparisons were made relative to the corresponding control groups. Sample sizes were established using power analyses based on effect sizes estimated from preliminary experiments. Following histotripsy, mice underwent post-treatment MRI to assess targeting accuracy and acute effects.

### 2.4. Histotripsy Ablation and Tumor Growth Assessment

Histotripsy ablation and subsequent tumor growth were monitored via a T2-weighted fast spin echo MRI sequence, as this sequence has been shown to effectively depict solid tumors and histotripsy ablation effects [19].

MRI volumes were quantified using a custom-written MATLAB script as described by Duclos et al. [18]. Background pixel intensities (PIs) of each image sequence were calculated by manually placing a circle with a 1 mm radius in the contralateral (untreated) side of the brain. PIs within each circle were averaged to obtain the background signal. Hyper/hypointense pixels were defined as those that were at least +/− 3 standard deviations outside of the background mean pixel intensity. Hyper/hypointense volumes were then calculated by multiplying the number of hyper/hypointense pixels by the voxel volume (in-plane resolution = 0.001 mm^2^/pix; slice thickness = 1 mm). In the brain, hyperintense regions are caused by prolonged T2 relaxation times from edema, inflammation, or tumor tissue [25], while hypointense regions indicate areas with high iron content associated with histotripsy ablation [19]. Post-treatment hypointense regions on T2-weighted MRI were defined as the ablation volume. Ablation volumes were normalized by dividing the post-treatment ablation volume by the pretreatment hyperintense regions on T2-weighted MRI (tumor) to account for any variation in tumor size between groups.

Mice were imaged with MRI prior to histotripsy, immediately following histotripsy, 1–3 days after histotripsy, and weekly until mice reached their survival endpoint, which was defined as a weight loss of >20% of maximum body weight throughout the study or indications of a moribund state (Figure 1B). Due to limitations in MRI-scanner availability, time points for tumor volume analysis were binned into weeks with respect to the day of tumor implantations as follows: week 2 (day 15–17 post-tumor inoculation), week 3 (day 19–23), and week 4 (day 26–28).

### 2.5. Endpoint Assessment

Mice were visually monitored every other day for the first week following histotripsy, then every day until the survival endpoint. Body weight and behavior (movement and posture) were recorded. All mice were euthanized with CO_2_ asphyxiation due to body weight loss.

### 2.6. Histological Assessment

After euthanasia, the skull was partially opened along the midline, and the brain and skull were submerged in 10% formalin for 24 to 48 h. Fixed brains were then extracted from the skull, embedded in paraffin blocks, and cut into 6 μm sections parallel to the direction of ultrasound propagation (coronal sections of the mouse brain). Tissue embedding and hematoxylin and eosin (H&E) staining were completed at the University of Michigan Tissue and Molecular Pathology Shared Resource core (iLab, University of Michigan, Ann Arbor, MI, USA). Blinded histological assessment was performed by a board-certified pathologist.

### 2.7. Statistical Analysis

Statistical analyses were performed in GraphPad Prism 10 and RStudio version 2024.09.1+394. Control and treated initial pretreatment tumor volumes were compared with unpaired *t*-tests to discern any differences in initial tumor volume between groups at the time of treatment. Ablation volumes were compared using a one-way ANOVA across treatment levels, followed by Tukey’s multiple comparisons test. Tumor volume data were grouped into pre-defined post-implantation time bins and were compared using a two-way ANOVA, with treatment group and time point treated as fixed factors. Repeated measures from the same animals across time points were accounted for using a matching-rows design. Post hoc multiple comparisons were performed by comparing all treatment groups within each time bin using Tukey’s multiple-comparisons test. Kaplan–Meier survival curves were compared with a log-rank (Mantel–Cox) test. A *p*-value of <0.05 was considered statistically significant. No data points were excluded from statistical analyses for any mouse at any time point. Mice were imaged, weighed, and observed in a random order for each time point to minimize potential confounders.

## 3. Results

### 3.1. Ablation Volumes

**Figure 2:**
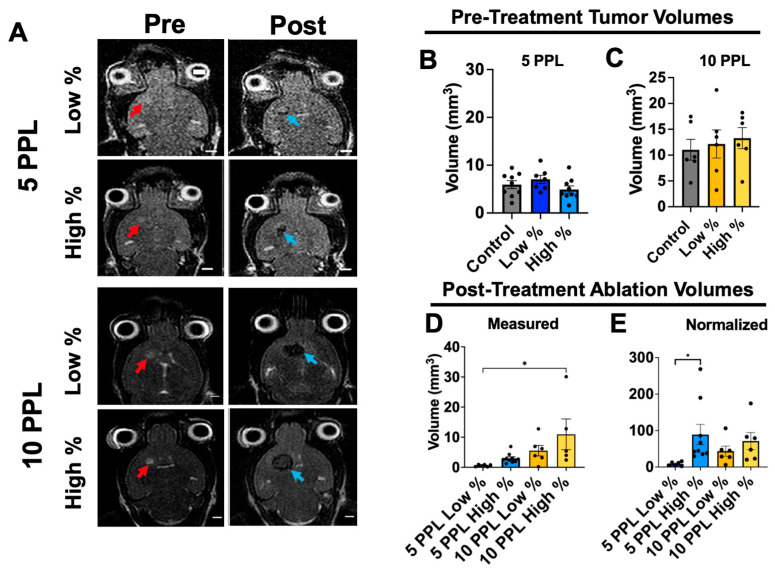
(**A**) Pre- vs. post-histotripsy T2-weighted MR images. Tumors were depicted as hyper-intense regions (red arrows) in pre-treatment images. Histotripsy ablation zones were depicted as hypointense regions (blue arrows) in post-treatment images. Scale bars = 2 mm. (**B**,**C**) Initial pre-treatment tumor volumes for 5 and 10 PPL experiments. There were no statistical differences in initial tumor volumes as measured from pre-treatment T2-weighted MRI amongst 5 PPL groups and amongst 10 PPL groups. (**D**,**E**) Measured and normalized ablation volumes. Ablation zones measured from post-treatment T2-weighted MRI increased with histotripsy treatment level. Ablation zones normalized to pre-treatment tumor volumes (ablation volume normalization coefficient) also increased with histotripsy treatment level. Scale bars = 2 mm. * *p* < 0.05.

Tumors were depicted on T2-weighted MRI as well-contained hyperintense regions prior to histotripsy (Figure 2A). Initial tumor volumes did not significantly vary between treatment levels within either group and were as follows: 5.9 ± 2.46, 7.03 ± 2.26 (*p* = 0.58 compared to control), and 4.92 ± 2.30 mm^3^ (*p *= 0.87) for 5 PPL control, low-percent, and high-percent groups, respectively, and 11.03 ± 4.95, 12.15 ± 6.69 (*p* = 0.93), and 13.28 ± 5.01 mm^3^ (*p* = 0.99) for 10 PPL control, low-percent, and high-percent groups, respectively (Figure 2B,C). The overall average initial tumor volume, weighted by group sample size, was significantly smaller for 5 PPL groups at the time of treatment compared to that of the 10 PPL groups (5.88 mm^3^ versus 12.15 mm^3^, respectively; *p* < 0.01).

Ablation regions were depicted as hypointense areas on T2-weighted MRI approximately 10 to 20 min after histotripsy (Figure 2A). Ablation volumes measured from post-treatment T2-weighted MRI generally increased with treatment level and were 0.6 ± 0.4, 2.3 ± 1.6, 5.5 ± 4.3, and 10.9 ± 11.4 mm^3^ for 5 PPL low-percent, 5 PPL high-percent, 10 PPL low-percent, and 10 PPL high-percent groups, respectively (Figure 2D). The 10 PPL high-percent ablation volumes were significantly higher than those of the 5 PPL low-percent treatment level (*p* = 0.02). Although the measure ablation volumes did not significantly vary between other groups, there was a trend in increased ablation volume with treatment level.

Ablation volumes were normalized with pre-treatment tumor volumes to account for variation in initial tumor volume (Figure 2E). The 5 PPL high-percent treatment level had the highest normalized ablation volume with 89.0 ± 83.7%, compared to 8.7 ± 5.3% of the 5 PPL low-percent treatment level. The 10 PPL low-percent and high-percent treatment levels had 43.5 ± 34.2% and 71.3 ± 57.2% normalized ablation volumes, respectively. The normalized ablation volumes differed from the targeted 25% or 75% of the tumor volumes for both the 5 and 10 PPL treatment levels; this was due to either blooming artifacts from blood products on post-treatment MRI [26] or inconsistent cavitation events due to skull aberrations.

### 3.2. Tumor Progression

**Figure 3:**
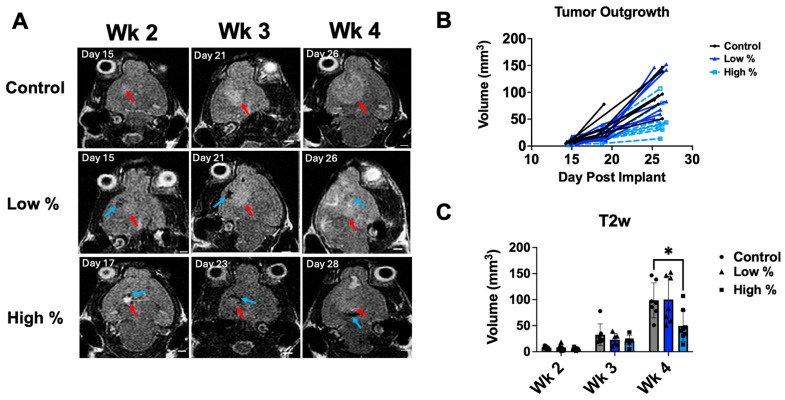
Weekly MRI for 5 PPL treatment levels. (**A**) Representative T2-weighted MR images for weeks 2–4 following intracranial tumor implantation, with red arrows indicating hyperintense/tumor regions and blue arrows indicating hypointense/ablation regions. Scale bars = 2 mm. (**B**) Tumor outgrowth curves for control, low-percent, and high-percent treatment groups as a function of days post-tumor implantation. (**C**) Binned hyperintense tumor volumes as measured from T2-weighted MRI. High-percent treatment resulted in significantly decreased tumor volume at Week 4. Dark blue bars indicate low-percent groups and light blue bars indicate high-percent groups. Histotripsy was performed at days 12–14 after tumor implantation. * *p* < 0.05.

**Figure 4:**
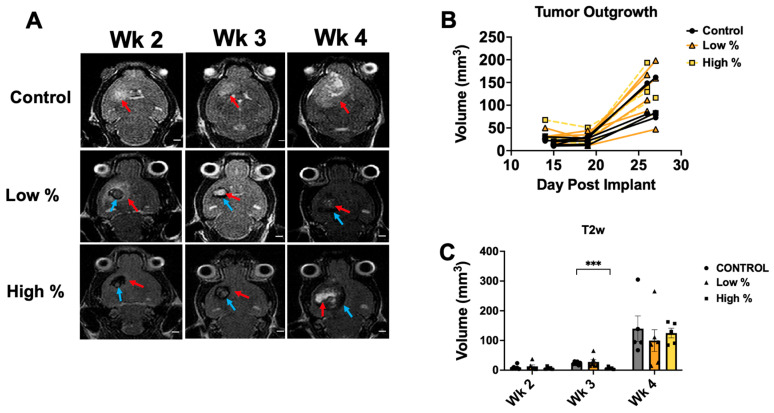
Weekly MRI for 10 PPL treatment levels. (**A**) Representative T2-weighted MR images for weeks 2–4 following intracranial tumor implantation, with red arrows indicating hyperintense/tumor regions and blue arrows indicating hypointense/ablation regions. Scale bars = 2 mm. (**B**) Tumor outgrowth curves for control, low-percent, and high-percent treatment groups as a function of days post-tumor implantation. (**C**) Binned hyperintense tumor volumes as measured from T2-weighted MRI for control, low-percent, and high-percent treatment groups. Histotripsy was performed on day 12 after tumor implantation. Abbreviation: wk, week. *** *p* < 0.001.

Tumor progression was monitored with T2-weighted MRI at weeks 2, 3, and 4 after tumor implantation for all groups (Figure 3 and Figure 4). Control tumors in the 5 PPL experiment showed exponential growth with well-defined hyperintense boundaries at week 4, as depicted by MRI (Figure 3A,B). Five PPL low-percent treated tumors also displayed exponential growth over weeks 2–4; yet the treated tumor region at week 4 had a discontinuous, inhomogeneous appearance inside the tumor, likely due to the ablation, while control tumors had a homogeneous appearance. The 5 PPL high-percent treatment tumors had significantly lower tumor volumes at week 4 compared to those of the control group (*p* < 0.03) (Figure 3C).

Control tumors in the 10 PPL experiment similarly displayed exponential growth over weeks 2–4, with a high degree of hyperintense necrosis in the tumor core at week 4 (Figure 4A). The 10 PPL low-percent treatment tumors were depicted as a relative hypointense core at week 2 with a hyperintense boundary, followed by a hyperintense core with hypointense boundaries at weeks 3–4 (Figure 4A). Additionally, 10 PPL high-percent treatment tumors showed substantial hypointensity at weeks 2–3, followed by a hyperintense tumor core at week 4 (Figure 4A). Tumor outgrowth curves showed slow growth through day 19, with increased growth up to day 27 for all groups (Figure 4B). The 10 PPL high-percent treatment tumor volumes were significantly lower at week 3 compared to controls (*p* < 0.01) but recovered to control tumor volumes by week 4 (Figure 4C). Hyperintense volumes measured from T2-weighted MRI for all groups are summarized in Table 1.

### 3.3. Survival Analysis

**Figure 5:**
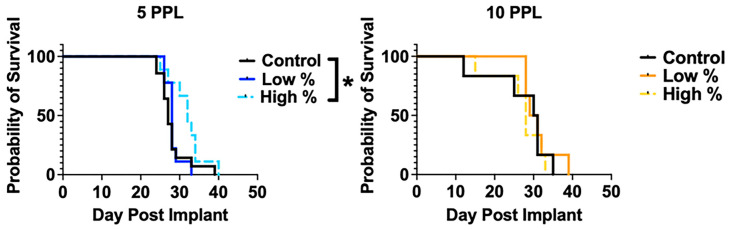
Kaplan–Meier survival curves for all levels of histotripsy. The 5 PPL high % group showed significantly enhanced survival with a 5-day (18.5%) median survival benefit compared to untreated controls (*p* = 0.04). * *p* < 0.05.

Kaplan–Meier survival curves and corresponding statistical data are shown in Figure 5 and Table 2, respectively. Median survival for low-percent and high-percent treatment levels within the 5 PPL experiment was 28 (*p* = 0.59) and 32 (*p* = 0.04) days, respectively, compared to 27 days for the control group. The 5 PPL high-percent treatment resulted in a significantly prolonged median survival of 5 days (18.5%) (*p* = 0.04). There were no significant differences between any of the groups in the 10 PPL experiment. The median survival was 30.5, 30, and 28 days for the control, low-percent (*p* = 0.58), and high-percent (*p* = 0.70) treatment groups, respectively.

### 3.4. Descriptive Histological Assessment at Endpoint

**Figure 6:**
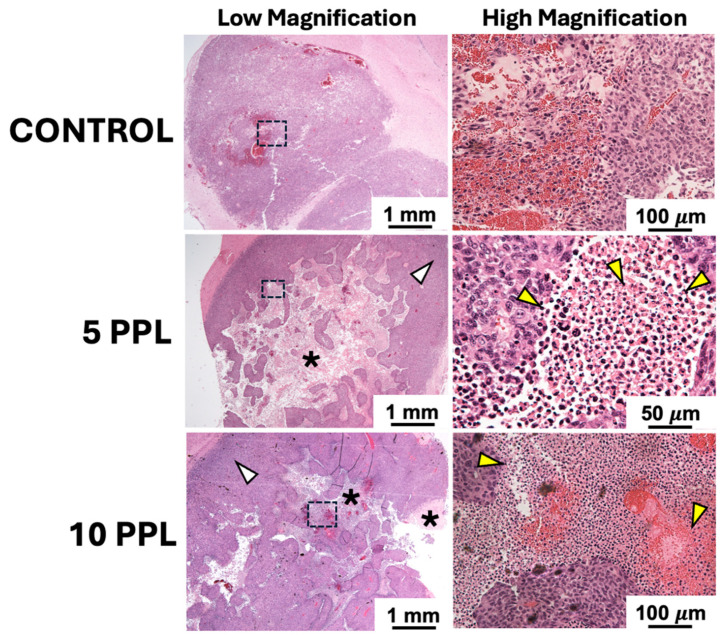
Representative H&E staining at the study endpoint for control, 5 PPL high-percent, and 10 PPL high-percent groups at low magnification (2×) and high magnification (20–40×). Both the 5 PPL and 10 PPL high-percent treatment groups exhibited visible areas of cellular disruption in the tumor core from histotripsy (asterisks). The 5 PPL high-percent group demonstrated more complete tissue homogenization, including disrupted cell nuclei (yellow arrows), compared to the 10 PPL high-percent group. Both treatment groups showed outward tumor cell proliferation at the time of euthanasia (white arrows). Black dashed boxes indicate areas shown at higher magnification.

H&E staining showed hypercellular growth for the untreated control tumors at endpoint, with pockets of intrinsic hemosiderin deposition and necrosis in the tumor core (Figure 6). Both the 5 PPL and 10 PPL high-percent treatment groups had visible regions of cellular disruption, presumably due to histotripsy treatment within the tumor core. The 5 PPL high-percent group showed more complete tissue homogenization in the form of disrupted cell nuclei within the tumor core compared to the 10 PPL high-percent group; however, both treatment levels resulted in outward tumor cell proliferation at the time of euthanasia. Within the treatment region of the 10 PPL high-percent group, there appeared to be pockets of hemosiderin, potentially caused by disrupted blood vessels in the tumor from histotripsy.

## 4. Discussion

Recently, histotripsy has shown positive clinical outcomes in the cancer realm [27] and has been gaining increasing attention for its ability to noninvasively and precisely ablate solid tumors. However, brain tumors present a unique challenge for histotripsy due to the high degree of attenuation and aberration induced by the presence of the skull, reducing the efficiency of ultrasound transmission to the brain. Moreover, primary malignant brain tumors like GBM are highly vascularized and aggressive, resulting in an extremely challenging pathology for any stand-alone treatment modality. To the authors’ knowledge, the present study is the first to investigate the survival effects of transcranial histotripsy for brain tumor treatment.

This study examined the effect of two levels of histotripsy (5 PPL and 10 PPL) with either a low (~25%) or high (~75%) volume of the tumor targeted using a custom-built 2 MHz custom-built transducer. Our results showed that a one-time histotripsy treatment applied at 5 PPL targeting a higher percentage of the tumor produced significant survival prolongation of 5 days compared to untreated controls in a highly aggressive murine GBM model (GL261 cells embedded in C56BL/6 mouse brain). The survival extension observed here is comparable to that reported in prior studies evaluating surgical tumor resection in the GL261 model [26]. There were no significant differences in survival times for the 5 PPL low-percent group, nor in either of the 10 PPL groups, compared to their respective untreated control groups. Our results indicate that the combination of dose and target tumor volume must be optimized for maximizing tumor cell kill while minimizing adverse effects, such as hemorrhage and edema caused by histotripsy [17]. All animals analyzed in this study tolerated the histotripsy well and showed no signs of distress. None of the treatment groups resulted in substantially decreased survival times compared to the untreated controls, indicating that the treatment levels tested in this study were feasible and safe.

MRI provides insight into the transient effects of transcranial histotripsy. Previous reports showed that, with T2-weighted MRI, the histotripsy boundary and resulting cellular homogenate appear hypointense, and the histotripsy-induced inter-lesion blood products appear acutely hypointense with transition to hyperintense during the first week, as the homogenate is absorbed by healthy tissue and the hemoglobin is metabolized [19]. This general pattern was corroborated in the present study. Immediately following histotripsy, the ablation volume was depicted as a hypointense region, which increased as the pulses per location and/or targeted volume increased. This result was expected and verified that a higher degree of energy, resulting in higher levels of acute tissue damage, was delivered to the tumor during the application of higher treatment levels (i.e., 10 PPL).

The lowest level of treatment (5 PPL low percent) resulted in outward T2-weighted hyperintense tumor growth after histotripsy, while the group that resulted in significant survival benefit (5 PPL high percent) displayed minimal hyperintense tumor regrowth over weeks 2–4. This may be because a higher portion of peripheral tumor cells were killed with the higher-treatment levels, resulting in slower outward growth and longer overall survival. However, it must be noted that normalized ablation volumes were comparable across the high-percent groups for 5 PPL and 10 PPL (Figure 2E), indicating that therapeutic outcome does not appear to scale directly with ablation volume alone. This suggests that differences in lesion morphology or downstream biological responses may also contribute to the observed survival benefit in addition to a higher proportion of tumor cell death. Additionally, striking a balance between sufficient tumor cell kill and preserving a fraction of intact tumor cells may be important for promoting anti-tumor immune responses, including T-cell recruitment, interferon-gamma production, and reductions in myeloid-derived suppressor cell populations [28]. However, as evidenced by endpoint tissue histology, outward tumor regrowth was seen in both the low-percent and high-percent treatment levels, indicating that further studies to optimize the treatment regimen are needed to more effectively reduce subsequent tumor regrowth, especially at the periphery, where tumor cells are highly prolific [28].

Neither of the 10 PPL treatment groups resulted in increased survival times compared to controls. The reason for this may be twofold. First, a more aggressive treatment regimen may induce a higher degree of blood products in the lesion and subsequent brain edema, offsetting any acute beneficial effects of histotripsy-induced tumor cell death. This is evidenced by the large hypointense volumes on T2-weighted MRI at week 4 for the 10 PPL groups, which may be caused by susceptibility effects that result from iron deposition from red blood cell lysis after brain trauma [29,30]. Second, the average tumor volume for the 10 PPL treatment groups at the time of treatment was larger than that of the 5 PPL treatment groups (*p *= 0.02). GL261 tumors undergo histopathological changes in the form of proliferation, vascularization, and development of necrotic regions as they progress [31], potentially rendering a one-time histotripsy treatment ineffective for higher-tumor volumes, even at a higher PPL. This result is corroborated in the clinic, as evidence suggests a strong correlation between high-preoperative tumor volume measured from contrast-enhanced MRI and shortened survival due to a lesser extent of resection and higher residual tumor volumes after surgery [32,33,34].

The GL261 GBM model used in this study is a widely used immunocompetent model for studying GBM, due to its fast and highly reproducible tumor growth [35,36]. However, its aggressive tumor growth makes it extremely challenging to induce any separation in treatment groups. Even if histotripsy produces a therapeutic effect in the initial days following treatment, the exponential growth of the tumor seemingly overwhelms any effects immunological responses may have and results in a tumor that looks indistinguishable from the controls. This may explain the modest survival benefit incurred by the single histotripsy treatment investigated in this study. Future studies should investigate the effect of histotripsy on other GBM models to discern model-dependent effects. Additionally, multiple histotripsy treatments may be required to achieve a clinically relevant survival extension of >30%, similar to reports for temozolomide in the GL261 model [37]. Evaluating repeated histotripsy sessions will be the focus of future studies.

### Limitations

There are several limitations to the present study. Potential batch effects were reduced to the best of our ability by including tumor-bearing, untreated mice from each individual experimental group in the respective control groups used for comparisons in this study. There was no method of real-time feedback for localizing the cavitation cloud or confirming cavitation for each transmitted pulse during each treatment. This may have led to variations in the received histotripsy dose to the brain within groups due to potential mistargeting of the cavitation cloud during treatments. The lack of real-time cavitation feedback was addressed with MRI-based stereotactic alignment to ensure consistent treatment across animals, as well as immediate post-histotripsy MR imaging to confirm treatment efficacy and targeting. Real-time feedback on the cavitation level is a focus for future preclinical studies. It must be noted that the lack of cavitation-based feedback is only a limitation of preclinical transcranial histotripsy experiments; human transcranial histotripsy treatment has been developed with real-time cavitation mapping for accurate targeting [16]. Despite these limitations, this study provides a foundation on which to build future research for clinical translation of transcranial histotripsy.

## 5. Conclusions

This study showed that a one-time transcranial histotripsy treatment can produce a mild survival benefit on its own compared to untreated controls. A higher number of treatment locations targeted within and surrounding the tumor is needed to produce prolonged survival times compared to a lower number of treatment locations.

Future studies are needed to further understand and optimize transcranial histotripsy in murine models, including more advanced treatment scanning patterns to minimize treatment time as well as real-time monitoring algorithms for ensuring complete and consistent homogenization of brain tumor tissue. Additionally, multifaceted treatment strategies that combine histotripsy with chemotherapy or immunotherapy using advanced instrumentation [38] may further enhance therapeutic benefit by combining tumor debulking with targeted elimination of residual tumor cells.

## Figures and Tables

**Table 1:** Hyperintense volumes from T2-weighted MRI.

Experiment	Treatment Level	N	# Locs	Week 2	Week 3	Week 4
**5 PPL**	Control	9	N/A	9.87 ± 1.38	26.3 ± 5.73	119 ± 44.2
	Low %	9	18 ± 6	19.0 ± 3.02	41.4 ± 6.47	129 ± 25.6
	High %	9	45 ± 5	8.37 ± 3.78	41.8 ± 19.6	46.1 ± 43.9
**10 PPL**	Control	6	N/A	9.40 ± 7.25	23.45 ± 5.23	139 ± 95.8
	Low %	6	24 ± 5	12.95 ± 12.6	27.7 ± 20.0	99.6 ± 90.0
	High %	6	73 ± 24	6.71 ± 4.56	6.64 ± 4.18	124 ± 36.4

**Table 2:** Survival outcomes.

Experiment	Treatment Level	N	Max. Survival (Days)	Median Survival (Days)	*p*-Value (Relative to Control)
**5 PPL**	Control	14	39	27	--
	Low %	9	33	28	0.59
	High %	9	40	32	0.04
**10 PPL**	Control	6	35	30.5	--
	Low %	6	39	30	0.58
	High %	6	33	28	0.70

## Data Availability

The datasets used and/or analyzed for the current study are available from the corresponding author upon reasonable request.

## Abbreviations

The following abbreviations are used in this manuscript:

BBB	Blood–brain barrier
GBM	Glioblastoma
H&E	Hematoxylin and eosin
IACUC	Institutional Animal Care Use Committee
MRI	Magnetic resonance imaging
OR	Operating room
PPL	Pulses per location
WK	Week
